# Effects of basic medical insurance integration on subjective wellbeing of residents in China: empirical evidence from a quasi-experiment

**DOI:** 10.3389/fpubh.2023.1211350

**Published:** 2023-08-16

**Authors:** Xin Na, Lingzhi Ding, Junxia Wang, Shuzhen Chu

**Affiliations:** School of International Pharmaceutical Business, China Pharmaceutical University, Nanjing, China

**Keywords:** basic medical insurance integration, the Urban and Rural Resident Basic Medical Insurance, urban and rural residents, subjective wellbeing, PSM-DID

## Abstract

**Introduction:**

Enhancing the wellbeing of residents through universal health coverage (UHC) is a long-term policy goal for China. In 2016, China integrated the New Rural Cooperative Medical Scheme (NRCMS) and the Urban Resident Basic Medical Insurance (URBMI) into the Urban and Rural Resident Basic Medical Insurance (URRBMI) to address the problem of fragmentation.

**Objective:**

The objective of this study was to investigate whether the integration of basic medical insurance had an impact on the subjective wellbeing of Chinese residents.

**Methods:**

Using the China Household Finance Survey data of 2015 and 2019, we empirically estimated the influence of the integration of basic medical insurance on Chinese residents through the difference-in-difference method based on propensity score matching (PSM-DID).

**Results:**

Our findings indicate that the integration of basic medical insurance improved the subjective wellbeing of the insured population. Additionally, through heterogeneity testing, we validated that the integration increased the subjective wellbeing of residents from less developed regions in West China and rural areas, as well as those with older adult dependents. However, the subjective wellbeing of low-income groups, who were expected to benefit more from the URRBMI, did not improve significantly, at least in the short term.

**Conclusion:**

According to our research, the integration of basic medical insurance in China supports the country's objective of achieving equality and providing universal benefits for its residents. The introduction of the URRBMI has had a positive impact on the subjective wellbeing of insured individuals. This is particularly beneficial for disadvantaged groups in less developed regions, as well as for residents with older adult dependents. However, the subjective wellbeing of the middle-income group has improved significantly, whereas that of the low-income group, despite being the intended beneficiaries of the integration, did not show significant improvement.

**Recommendations:**

From a funding perspective, we recommend establishing a dynamic adjustment funding system that links residents' medical insurance funding standards with their disposable income. Regarding the utilization of the URRBMI, the benefit packages should be expanded, particularly by covering more outpatient services through risk pooling. We call for further research with additional data and continued efforts on improving wellbeing of residents, particularly for disadvantaged populations.

## Introduction

China's basic medical insurance schemes cover 1,364,240,000 individuals which account for more than 95% of the Chinese population by the end of 2021,^1^ that makes the Chinese government aim to achieve universal health coverage (UHC), which is one of the biggest health insurance payers in the world (1). However, it took the Chinese government years of major effort to resolve the problem of fragmentation which contributes to the disparity in health benefit packages for urban and rural residents and impedes the progress in achieving UHC.

The basic medical insurance schemes of China for urban and rural residents had been developed separately, hence a relatively unfair healthcare system for a long time.

Since 1949, China has implemented a healthcare insurance system consisting of three separate programs—the Labor Insurance Program (LIP) for urban employees, the Government Insurance Program (GIP) for government staff, public institution employees, veterans, and university students, and the Cooperative Medical Scheme (CMS) for rural residents.

However, excessive utilization of healthcare funds by insured individuals led to the pilot of mandatory government-run basic medical care, replacing the LIP and GIP. In December 1998, the Urban Employees Basic Medical Insurance (UEBMI) was proposed nationwide.

At the same time, the marketization in economic system reform and opening-up led to the decline of the CMS. In January 2003, China proposed to establish the New Rural Cooperative Medical Scheme (NRCMS) for rural residents who were not eligible for the UEBMI. The NRCMS was designed as a remedy to disperse rural residents' risk of major diseases, reduce poverty due to illness, and increase rural residents' satisfaction with medical services (2). Implemented at a relatively low per-capita funding rate, the NRCMS effectively reduced the probability of rural Chinese households falling into poverty, as well as the risk of return to poverty caused by family members being hospitalized with illness (3).

Parallel to the exploration and establishment of basic healthcare insurance schemes for urban employees and rural residents was the lack of medical insurance coverage for urban residents. In 2007, China began to pilot the Urban Resident Basic Medical Insurance (URBMI) covering all non-employed urban residents, the funding system of which was similar to the NRCMS, but participants were required to contribute a greater share of the premium in comparison to rural residents; so far, China had ushered in a new era of universal healthcare.

However, the division between the NRCMS and the URBMI gradually led to institutional imbalances and a disparity in resources, since the Chinese basic healthcare insurance system was established for different groups of people with a dual-track structure of China's socioeconomic development instead of being built from the perspective of universal coverage in the first place. The NRCMS pooled at the county level had lower reimbursement rates and annual caps compared to the URBMI pooled at the municipality level, even though rural residents' contributions increased year after year (4). Take Jiangsu Province, located in Eastern China with upper-level economic development, as an example. In 2008, the average annual cap of Jiangsu Province for the residents covered by URBMI was ¥80,000, while for the residents covered by NRCMS was ¥70,000; meanwhile, the reimbursement rates of URBMI for hospitalization were higher than that of the NRCMS in the same district (5).

Therefore, the State Council issued a specific document in January 2016 requiring the merging of the basic medical insurance schemes for both urban and rural residents into Urban and Rural Resident Basic Medical Insurance (URRBMI) to achieve a fair UHC. It referred to the integration in six aspects mainly on the policy level, including coverage, financing policies, medical benefits, the directories of insured drugs, the management of designated hospitals, and the management of insurance funds (6).

The basic medical insurance integration has narrowed the gap between urban and rural medical security by bringing the URBMI and the NRCMS into the same system and consolidating the funds of the two systems into unified management. In particular, the scope of the directories of insured drugs and the management of designated hospitals have been expanded through the integration. On the one hand, before the implementation of the integration, the URBMI directory included more than 2,200 drugs, whereas the NRCMS directory included about 700–1,300 drugs (7). However, after the integration, the directories of insured drugs aligned with the URBMI directory, resulting in a significant increase in the number of drugs that can now be covered by medical insurance, which helped to alleviate the financial burden on patients and improve their access to treatment. On the other hand, after the integration, the management of insurance funds has transited from the county level to the municipal level, hence expanding the scope of medical treatment options, especially more designated hospitals for residents. For example, in Chengdu, the capital city of Sichuan province, the number of designated hospitals increased from 500 to 935 after the integration (8). Overall, the fairness of medical security for urban and rural residents has been improved, which motivated more people to participate in the URRBMI. It is shown that 87.359 million people took part in it in the year 2017, which is an increase of 424.99 million compared to the end of the previous year (9).

The economic boom over the past decades has allowed China to increase investments in healthcare so that the country's healthcare system has undergone significant changes. Despite tremendous socioeconomic development and an enhanced standard of living, the subjective wellbeing of Chinese residents, defined as their overall feeling of happiness in life or life satisfaction (10), remained unsatisfactory. The levels initially declined sharply during rapid economic growth and then experienced a slight recovery (11). To put it differently, in the year of the implementation of the basic medical insurance integration, the United Nations released its 2016 Global Happiness Index Report, which ranked mainland China 83rd out of 157 countries and regions (12).

Fluctuations in the pace of economic progress have led to heightened feelings of insecurity and inequality, thus negatively impacting the subjective wellbeing of residents. As China transitioned from centrally planned macroeconomic management to embracing free market principles and underwent changes in social welfare systems, great differences in welfare existed between urban and rural groups. These stark differences and the accelerated pace of life brought on by rapid economic development have resulted in great psychological pressure on residents (13). Therefore, policymakers need to find solutions to address these issues.

The integration is bound to have a significant influence on China's basic healthcare insurance system and the subjective wellbeing of billions of people across the country. Thus, using data from the China Household Finance Survey (CHFS), we take the integration as a quasi-natural experiment and apply the propensity score matching-difference-in-differences method (PSM-DID) to investigate whether the integration, regarded as a lever to facilitate the redress of insurance inequality, enhances the subjective wellbeing of the insured residents significantly.

## Literature review

### Effects of the basic medical insurance integration on residents

Our study contributes to the emerging literature on basic medical insurance integration in China, where several research projects have demonstrated the effects of the integration on residents, particularly on the vulnerable population.

Existing literature underlines the evaluation of how basic health insurance integration affects healthcare equity since China integrated the URBMI and the NRCMS into the URRBMI to reduce the persistent inequality in the basic medical insurance system. Some scholars find that the URRBMI plays a positive role in bridging the gap in health service utilization between rural and urban since the implementation of the integration improves the ability of basic medical insurance to withstand risks and gives rural residents improved access to health services (14). While others hold that the integration does not narrow gaps in total and out-of-pocket medical expenditure, as well as reimbursement between rural and urban residents, this result may attribute to urban–rural inequality in access to healthcare and provincial fiscal spending on medical (15).

The vulnerable population draws attention from scholars in studies of basic medical insurance integration. The implementation of the URRBMI is found to facilitate the utilization of healthcare services for low-income rural residents, as well as relieve the medical burden of middle-income, middle-aged, and older adult residents from rural areas, but it cannot share the pressure of medical treatment for families having catastrophic health expenditure (16, 17). As for middle-aged and older adult residents from rural areas, the integration also effectively alleviates the loss of mobility of them, since the benefit package and reimbursement rate are enhanced so that these residents can seek medical care timely without concern of financial burden (4). Meanwhile, scholars are concerned about the effect of basic healthcare insurance integration on migrants, whose utilization of medical services and consumption are found to be improved by the URRBMI (18, 19).

It is noteworthy that, due to the comprehensive effects of basic medical insurance integration, some scholars are concerned that the integration affects residents' quality of life not only from physical and financial aspects but also from the mental aspect, so they introduce the concept of subjective wellbeing into their studies but the subjects still are a vulnerable population. An existing study proves that participating in the URRBMI significantly improved the subjective wellbeing of rural residents by lightening their healthcare burden, improving their access to medical care, and improving their health expectations, especially for the rural older adult, whose positive affection is improved significantly (20).

### Factors influencing subjective wellbeing

Subjective wellbeing is an overall emotional and cognitive evaluation of the quality of one's life (21), it is capturing the interest of more and more scholars and policymakers and has become a multidisciplinary research topic, involving psychology, sociology, and public health.

Existing studies on subjective wellbeing focus on the influencing factors involving personal characteristics, household features, social development, and spatial differences. As for personal characteristics, scholars consider age, gender, educational background, health conditions, and marriage status as major factors for subjective wellbeing (22–24). Referring to household features, such as fixed assets, housing conditions, aged dependents, and family expenditures, influence subjective wellbeing (25, 26). Regarding social development, existing studies find that the unemployment rate, social fairness, and policy institutions, such as policy beliefs and political freedoms, also affect subjective wellbeing (24, 27, 28). In respective of spatial differences, scholars carrying out research at the national scale and city scale hold that there are significant differences in subjective wellbeing in different regions (29, 30).

According to emerging studies, the overall effects of basic medical insurance integration on residents vary among the population, and vulnerable groups interest scholars the most. To the best of our knowledge, this study is the first study to document the causal effect of basic medical insurance integration on the subjective wellbeing of the general population.

## Data and methods

### Data source

We use publicly available data from the China Household Finance Survey (CHFS). The CHFS is a nationally representative and longitudinal survey conducted biannually since 2011 by the Survey and Research Center for China Household Finance of Southwest University of Finance and Economics. A stratified, three-stage, and probability proportional to size (PPS) sampling method is adopted in the CHFS that samples 40,011 households and 127,012 individuals distributed in 29 provinces, 367 counties (districts and county-level cities), and 1,481 communities. Furthermore, the CHFS collects information on demographic characteristics, social security and insurance, employment, income and consumption, and so on (31), and this abundant material provides data support for the empirical analysis of this study.

For our main analysis, we exclude individuals with missing values and use a sample of 12,654 residents who have complete data on insurance type in both CHFS2015 and CHFS2019. Like any major reform in China, the implementation of the integration varied across provinces, Chongqing Municipality, Shandong Province, Guangdong Province, and many places around the country, had been exploring means of institutional unification of the two medical insurance schemes before the State Council announced the policy in January 2016 (6), so we exclude the samples that had been covered by URRBMI in 2015 to ensure the accurate setting of the experimental group. To make the analysis more rigorous, we also exclude the individuals whose insurance type does not match their hukou status. Hukou is a legal document that identifies a person as a permanent resident of an area in China, and whether one is entitled to basic medical insurance programs for urban residents, i.e., URBMI, or for rural residents, i.e., NRCMS, is according to his or her hukou status.

### Description of variables

#### The core explained variable

Subjective wellbeing is utilized as the core explained variable. In the CHFS, respondents reported their perceptions of wellbeing on a five-point scale, ranging from 0 (very unhappy) to 4 (very happy), and the corresponding question is: “in general, do you consider yourself happy now?” This primary scale of subjective wellbeing measurement scale is widely used in major social surveys in China, such as the Chinese General Social Survey (32).

#### The core explanatory variables

The type of basic medical insurance that individuals are covered by is involved as the core explanatory variable in our study, with a value of 1 assigned to the URBMI, 2 to the NRCMS, and 3 to the URRBMI.

#### Control variables

In light of the existing literature, we group the control variables that might influence an individual's subjective wellbeing in three respects. As for the individual, we control variables for gender, age, education level, hukou, whether a party member, marital status, and health status. With regard to the household, we choose whether the household owns a house, the proportion of household members aged 60 years and older, household size, and total household income (taking its logarithm) as the potential factors of residents' subjective wellbeing. Concerning the region, whether from urban or rural area is controlled, we control regional dummy variables for each province (autonomous region/municipality directly under the central government) as a way to avoid the omitted variables bias caused by differences among provinces (22–24, 33–36). The results of descriptive statistical analysis for each category of variables are shown in Table 1.

**Table 1:** Variable definitions and summary statistics.

**Variables**	**Description**	***N***	**Mean**	**Min**	**Max**
Subjective wellbeing	Individuals' subjective perceptions of wellbeing, assigning values 0–4	12,654	2.75	0	4
	0 (worst) (1.37%)				
	1 (5.75%)				
	2 (30.37%)				
	3 (41.54%)				
	4 (best) (20.97%)				
Insurance	URBMI = 1 (4.43%)	12,654	2.00	1	3
	NRCMS = 2 (91.12%)				
	URRBMI = 3 (4.45%)				
Age	Continuous variables	12,654	57.45	19	96
Gender	Male = 1 (15.16%)	12,654	0.85	0	1
	Female = 0 (84.84%)				
Education level	Elementary school and illiterate = 1 (49.38%)	12,654	1.52	1	4
	Middle school or high school = 2 (49.35%)				
	Bachelor's degree = 3 (1.26%)				
	Master's degree and above = 4 (0.02%)				
Political organization membership	Political party members = 1 (88.44%)	12,654	0.12	0	1
	Not affiliated to any political organizations = 0 (11.56%)				
Hukou	Rural = 1 (95.03%)	12,654	0.95	0	1
	Urban = 0 (4.97%)				
Marriage status	Married = 1 (88.89%)	12,654	0.89	0	1
	Unmarried = 0 (11.11%)				
Self-assessment of health	From very unhealthy to very healthy, assigning a value of 0–4	12,654	2.14	0	4
	0 (worst) (5.25%)				
	1 (19.84%)				
	2 (40.41%)				
	3 (24.91%)				
	4 (9.59%)				
Household size	Continuous variables	12,654	6.87	1	39
Proportion of the population aged 60 years and older	Continuous variables	12,654	0.22	0	1
Household income	Continuous variables	12,654	7.79	−13.37	14.04
Employment status	Employed = 1 (77.86%)	12,654	0.78	0	1
	Unemployed = 0 (22.14%)				
Residence	Urban = 1 (32.46%)	12,654	0.68	0	1
	Rural = 0 (67.54%)				
Region	East = 1 (28.62%)	12,654	2.30	1	4
	Central = 2 (23.66%)				
	West = 3 (37.31%)				
	Northeast = 4 (10.41%)				

The proportions of the respondents of different characteristics are reported in brackets except for continuous variables.

### Model setting

#### Ordinary least squares (OLS) and ordered probit (oprobit) regression models

The core explained variable, subjective wellbeing, is discrete ordinal, so it is appropriate to use the ordered probit (oprobit) model. However, when the sample size is large, there is no difference between the results estimated using both the ordinary least squares (OLS) and the oprobit models, and the OLS model yields more intuitive and economically meaningful estimates (25, 37–39). Therefore, we apply the OLS model to estimate the effect of integration on residents' subjective wellbeing in regression of the following form (Equation 1), while the estimated results of the oprobit regression model are reported as evidence of the OLS regression.


(1) $$\begin{array}{l} {Subjective\;well-being}_{i}=a+\beta insurance+\gamma_{i}x_{i}+u_{i} \end{array}$$

In Equation 1, the explained variable is the *subjective well*−*being*_*i*_ of the individual, the core explanatory variable *insurance* is whether the individual is covered by the URRBMI, *x*_*i*_ is a control variable for other individual characteristics, *u*_*i*_ is a random disturbance term, and *i* in the equation represents the individual.

#### Difference-in-difference based on propensity score matching

The reform of basic medical insurance integration in China was guided by the government; meanwhile, if we only use the OLS regression model described above, it may result in self-selection-based endogeneity bias, so the difference-in-difference (DID) design, a quasi-experimental research, is conducted in our study, which is considered as an effective way to learn about causal relationships for implementation of public health policy (40). To deal with the endogeneity issue, we further use the propensity score matching method (PSM) to match similar samples based on individual characteristics to reduce selection bias, and the successfully matched samples are processed by the DID method to reduce the confounding effect caused by unobservable variables and estimate the average treatment effect of the policy. The DID model can be presented with Equation 2.


(2) $$\begin{array}{l} \begin{array}{l} Subjective\;well-being_{it}=\beta_{0}+\beta_{1}did_{it}+\beta_{2}post_{it} \end{array}\\ \;\begin{array}{l} +\beta_{3}treat_{it}+a_{i}control_{it}+\varepsilon_{it} \end{array} \end{array}$$

The effect of the integration of the basic healthcare insurances is estimated by comparing the differences between two changes in outcomes: (i) changes between the pre- and post-integration periods within the intervention group (individuals who changed insurance types, with a sample size of 563) and (ii) the pre- and post-integration periods in the control group (individuals who did not change insurance types, with a sample size of 12,091) (41). In Equation 2, *Subjective well*−*being*_*it*_ is the outcome (discussed below) for individual *i* measured in year *t*. The variables *treat*_*it*_ is binary such that *treat*_*it*_=1 if the individual is from the intervention group covered by NRCMS or URBMI in 2015 and switches to URRBMI in 2019, and *treat*_*it*_=0 if the individual if from the control group that does not switch the insurance type. *Post*_*it*_=1 for 2019 and 0 for 2015. *Did*_*it*_, the core explanatory variable, is the interaction term that equals *treat*_*it*_×*post*_*it*_. *Control*_*it*_ denotes the control variable; ε_*it*_ denotes the random error term.

β_1_ is the difference-in-differences estimator which reflects the net effect of 2016 integration on individual's subjective wellbeing, and if the integration improves residents' wellbeing, we expect β_1_ to be positive. β_2_ captures the simple pre-post change in the outcome. β_3_ captures the difference in the outcome between the intervention group and the control group.

## Results

### Descriptive analysis

The descriptive statistics in Table 2 show that a majority of included residents are middle-aged, male, educated to below high school, married, from the central region of China, and not affiliated with any political organizations, and most residents' self-assessed health is not good enough. Referring to supporting family, most respondents are employed and do not have family members aged 60 years and older.

**Table 2:** Baseline regression results of the OLS and the oprobit.

**Variable**	**(1) OLS**	**(2) Oprobit**
Insurance	0.2008^***^	0.2716^***^
	(0.0321)	(0.0435)
Age	0.0097^***^	0.0122^***^
	(0.0009)	(0.0009)
Gender	−0.0169	−0.0203
	(0.0241)	(0.0296)
Education level	0.0182	0.0170
	(0.0160)	(0.0199)
Political organization membership	0.0917^***^	0.1134^***^
	(0.0228)	(0.0295)
Hukou	−0.0974^**^	−0.1412^**^
	(0.0445)	(0.0584)
Marriage status	0.1266^***^	0.1472^***^
	(0.0289)	(0.0353)
Self-assess health	0.1851^***^	0.2298^***^
	(0.0086)	(0.0110)
Household size	0.0075^***^	0.0094^***^
	(0.0010)	(0.0012)
Proportion of population aged 60 years and older	0.2379^***^	0.3043^***^
	(0.0297)	(0.0378)
Household income	0.0129^***^	0.0159^***^
	(0.0025)	(0.0030)
Employment status	0.0552^**^	0.0658^**^
	(0.0215)	(0.0264)
Residence	0.0144	0.0206
	(0.0173)	(0.0215)
Region	0.0319^***^	0.0398^***^
	(0.0076)	(0.0096)
_cons	1.0195^***^	
	(0.0923)	
*N*	12,654	12,654
*R*^2^/Pseudo *R*^2^	0.0780	0.0318
*F*-value/Wald test value	70.61	897.49

Significance levels ^*^p <0.1; ^**^p <0.05; ^***^p <0.01.

### Baseline model estimation

To investigate the effect of basic medical insurance integration on residents' subjective wellbeing, we conduct a baseline regression with *insurance* as the core explanatory variable first. Based on the baseline regression, we use the PSM-DID approach for causal inference to obtain the net effect of integration, as well as further test the heterogeneity of the model. In the end, we test the robustness of our model.

Table 2 reports the baseline regression results of the OLS model and the oprobit model, that the basic medical insurance integration has a positive effect which is significant at the 1% on improving residents' subjective wellbeing. Compared with the URBMI and the NRCMS, URRBMI improves residents' subjective wellbeing by 20.08%, and the oprobit model also yielded similar estimation results. Overall, the integration of basic medical insurance significantly improves the subjective wellbeing of those previously covered by the URBMI and the NRCMS. It is noteworthy that the higher the household income and the score of self-assess health one has, the higher score he or she gives to the evaluation of wellbeing, i.e., these factors also play a contributing role in improving subjective wellbeing.

### Inference for causal effects

As mentioned above, the regression results of OLS and oprobit may have endogenous disturbances. On the one hand, the individuals' abilities, characteristics, and other unobserved factors can lead to omitted variable bias. On the other hand, residents' subjective evaluation of wellbeing may also directly affect their choice of insurance, resulting in reverse causation. Therefore, we further use the PSM-DID method to avoid selection bias and infer the causal effects.

### Propensity score matching

**Figure 1:**
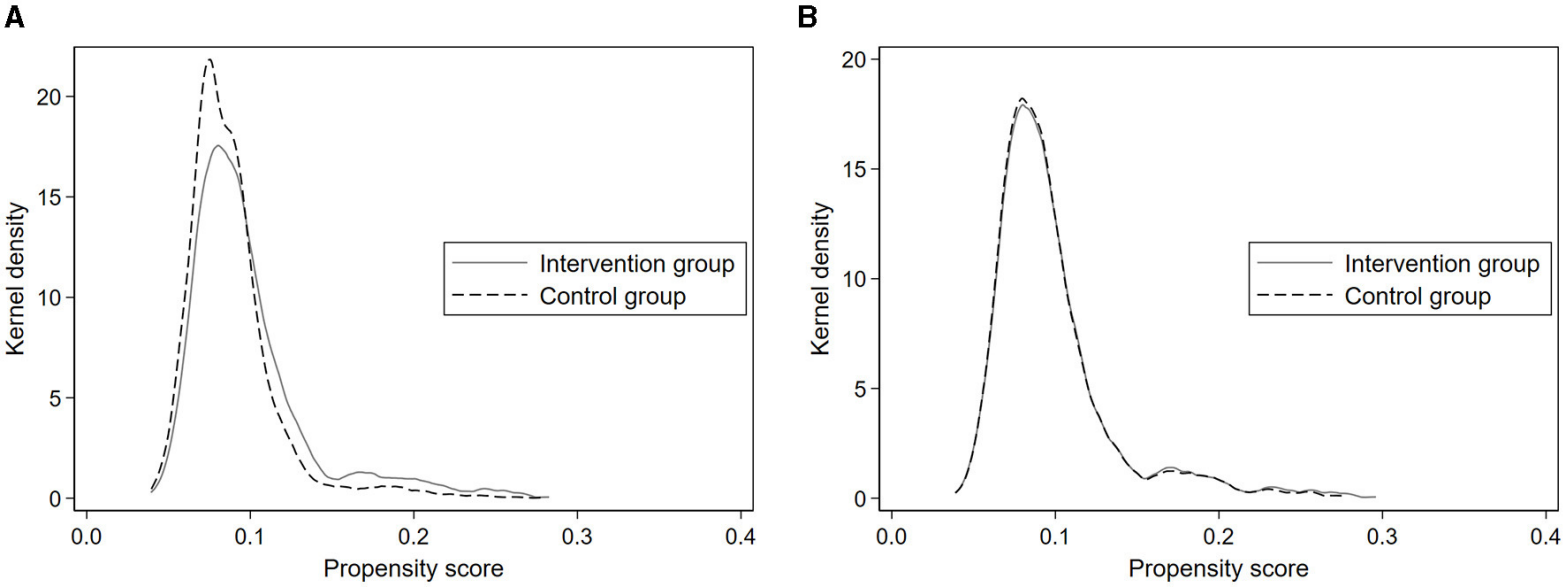
**(A)** The kernel density curves before PSM. **(B)** The kernel density curves after PSM.

Referring to the existing literature, we adopt 1:1 nearest neighbor matching and test for common trends and parallel trend assumption which are the two necessary preconditions. As shown in Figure 1A, when the control group is not matched to the intervention group, there is a large gap between the two kernel density curves. As shown in Figure 1B, however, after matching the curves almost overlapped, which proves that the characteristics of the intervention group and the control group are more consistent with the ideal matching effect.

As for the parallel trend assumption, the covariates of matched intervention and control groups should not differ significantly (42), and Table 3 shows the balance test result before and after the matching process, suggesting that the systematic differences among individuals between the intervention and control groups significantly reduce after using the matching method. After implementing the 1:1 nearest neighbor matching method, Table 3 shows that the absolute values of the standardized deviations of all covariates are <10%, as well as the *t*-test results of all covariates after matching do not have a significant difference between the intervention and control groups, indicating that the matching effect is ideal and satisfies the parallel trend assumption. To conclude, the matching method makes the intervention and control groups comparable.

**Table 3:** Balance test results.

**Covariates**	**Unmatched (U)/matched (M)**	**Intervention group**	**Control group**	**% deviation**	***T***	***P***
Age	U	58.855	57.309	12.9	4.16	0.000
	M	58.855	58.571	2.4	0.56	0.572
Gender	U	0.8055	0.85262	−12.5	−4.21	0.000
	M	0.8055	0.7913	3.8	0.84	0.401
Education level	U	1.4885	1.5222	−6.4	−2.06	0.040
	M	1.4885	1.5018	−2.5	−0.60	0.549
Political organization membership	U	0.09325	0.1178	−8.0	−2.46	0.014
	M	0.09325	0.09325	0.0	0.00	1.000
Hukou	U	0.89876	0.95533	−21.9	−8.36	0.000
	M	0.89876	0.89609	1.0	0.21	0.835
Marriage status	U	0.87744	0.89009	−3.9	−1.29	0.197
	M	0.87744	0.86234	4.7	1.06	0.287
Self-assess health	U	2.0995	2.1413	−4.2	−1.33	0.185
	M	2.0995	2.1155	−1.6	−0.37	0.708
Household size	U	6.492	6.9065	−5.0	−1.57	0.116
	M	6.492	6.2647	2.7	0.67	0.504
Proportion of population aged 60 years and older	U	0.25496	0.22117	9.5	3.14	0.002
	M	0.25496	0.24557	2.7	0.62	0.537
Household income	U	8.046	7.7625	9.4	2.80	0.005
	M	8.046	8.1039	−1.9	−0.50	0.617
Employment status	U	0.746	0.78184	−8.4	−2.76	0.006
	M	0.746	0.76021	−3.3	−0.78	0.434
Residence	U	0.59503	0.68338	−18.5	−6.05	0.000
	M	0.59503	0.59503	0.0	0.00	1.000
Region	U	2.3321	2.2916	4.3	1.30	0.192
	M	2.3331	2.3197	1.3	0.31	0.753

### Results of DID

Table 4 describes the results of DID analysis based on PSM, which determine the causal effects of basic medical insurance integration on residents' subjective wellbeing. There are no control variables included in Model (3), while DID with the covariates in Model (4) is employed to assess the effect of basic medical insurance integration. As shown in Table 4, all the coefficients of *did*_*it*_ are positive and statistically significant, even after controlling for the variables in respect of individual, household, and region in the Model (4). The regression results show that the integration of basic medical insurance can significantly increase the subjective wellbeing of individuals by 12.83% [*p* < 0.05, in Model (3)], while after the inclusion of control variables, the subjective wellbeing can be increased by 12.57% [*p* < 0.05, in Model (4)]. That is to say, the implementation of basic medical insurance integration has a significantly positive effect on the likelihood of improving residents' subjective wellbeing, therefore, our expectation is validated.

**Table 4:** Results of PSM-DID.

	**Model (3)**	**Model (4)**
	**Subjective wellbeing**	**Subjective wellbeing**
Treat	−0.0097	−0.0119
	(0.0394)	(0.0384)
Post	0.3015^***^	0.2666^***^
	(0.0164)	(0.0175)
Did	0.1283^**^	0.1257^**^
	(0.0564)	(0.0546)
Age		0.0087^***^
		(0.0009)
Gender		0.0057
		(0.0240)
Education level		−0.0007
		(0.0159)
Political organization membership		0.0939^***^
		(0.0225)
Hukou		0.0747^**^
		(0.0367)
Marriage status		0.1393^***^
		(0.0285)
Self-assess health		0.1880^***^
		(0.0085)
Household size		0.0013
		(0.0010)
Proportion of population aged 60 years and older		0.1743^***^
		(0.0297)
Household income		0.0097^***^
		(0.0025)
Employment status		0.0331
		(0.0213)
Residence		0.0171
		(0.0172)
Region		0.0300^***^
		(0.0076)
_cons	2.5941^***^	1.2714^***^
	(0.0115)	(0.0841)
*N*	12,640	12,640
*R* ^2^	0.031	0.096
Adj. *R*^2^	0.031	0.094

Significance levels ^*^p <0.1; ^**^p <0.05; ^***^p <0.01.

Table 4 also characterizes the association between influence factors and residents' subjective wellbeing. As observed from Table 4, the coefficients of all control variables are positive, especially in respect of individual, the political organization membership, marriage status and self-assess health can increase the subjective wellbeing of individuals notably by 9.39, 13.39, and18.80% [all *p* < 0.01, in Model (4)]. As for household respect, the proportion of population aged 60 years and older and household income also increase significantly (17.43%, *p* < 0.01; 0.97%, *p* < 0.01, respectively); and subjective wellbeing can be improved as well as by region by 3.00% (*p* < 0.01).

### Heterogeneity test

Region and household are potential dimensions associated with the effect of the basic medical insurance integration according to previous literature (15, 43), that the healthcare inequality attributed to uneven socioeconomic development across China influencing the improvement of residents' subjective wellbeing, hence, to assess the heterogeneity in the effects across regions and among different household characteristics groups is necessary.

### Heterogeneity test of regional characteristics

To assess the heterogeneity in the effects of basic medical insurance integration on individuals with different regional characteristics, the regressions are divided into urban and rural areas as well as East China, Central China, West China, and Northeast China samples for grouping.

Table 5 illustrates the differences in the effects of basic medical insurance integration on the subjective wellbeing of different groups located in urban and rural areas as well as in different regions of China. Models (5) and (6) are the results of heterogeneity for urban and rural individuals, as well as Models (7), (8), (9), and (10) for samples from different regions of China. First, the integration significantly enhances the subjective wellbeing of rural groups by 14.78% (*p* < 0.05) while it does not play a salient role in improving the wellbeing of urban groups. Second, the subjective wellbeing of individuals from West China groups is improved significantly by the integration by 18.30% (*p* < 0.05), while the integration also improves the subjective wellbeing of residents from East, Central, and Northeast China but in an insignificant way.

**Table 5:** Heterogeneity test results for regional characteristics.

	**(5)**	**(6)**	**(7)**	**(8)**	**(9)**	**(10)**
	**Rural**	**Urban**	**East**	**Middle**	**West**	**Northeast**
Treat	−0.0210	−0.0168	0.0454	−0.0207	−0.0184	0.1430
	(0.0515)	(0.0574)	(0.0809)	(0.0699)	(0.0596)	(0.1532)
Post	0.3145^***^	0.1627^***^	0.2266^***^	0.3609^***^	0.2759^***^	0.1502^***^
	(0.0213)	(0.0308)	(0.0320)	(0.0376)	(0.0289)	(0.0521)
Did	0.1478^**^	0.1249	0.0536	0.0303	0.1830^**^	0.1402
	(0.0717)	(0.0844)	(0.1235)	(0.0974)	(0.0849)	(0.1978)
Age	0.0086^***^	0.0087^***^	0.0055^***^	0.0094^***^	0.0090^***^	0.0132^***^
	(0.0011)	(0.0015)	(0.0017)	(0.0020)	(0.0014)	(0.0029)
Gender	−0.0115	0.0391	−0.0230	−0.0079	0.0173	0.0682
	(0.0312)	(0.0375)	(0.0477)	(0.0482)	(0.0381)	(0.0765)
Education level	0.0052	−0.0135	−0.0291	0.0438	−0.0233	0.0386
	(0.0196)	(0.0274)	(0.0301)	(0.0337)	(0.0260)	(0.0466)
Political organization membership	0.1173^***^	0.0371	0.1530^***^	0.0609	0.1141^***^	−0.0406
	(0.0271)	(0.0406)	(0.0408)	(0.0459)	(0.0379)	(0.0695)
Hukou	0.0137	0.0817^**^	0.0494	0.2200^***^	0.0315	0.0501
	(0.1227)	(0.0397)	(0.0762)	(0.0760)	(0.0588)	(0.0946)
Marriage status	0.1296^***^	0.1638^***^	0.1359^**^	0.1055^*^	0.1154^**^	0.3087^***^
	(0.0351)	(0.0486)	(0.0562)	(0.0576)	(0.0457)	(0.0862)
Self–assess health	0.1820^***^	0.2016^***^	0.2016^***^	0.1904^***^	0.1761^***^	0.1679^***^
	(0.0102)	(0.0151)	(0.0165)	(0.0178)	(0.0138)	(0.0241)
Household size	0.0004	0.0034^*^	0.0004	0.0001	0.0030^*^	0.0026
	(0.0013)	(0.0018)	(0.0019)	(0.0022)	(0.0017)	(0.0031)
Proportion of the population aged 60 years and older	0.1859^***^	0.1411^***^	0.1637^***^	0.1308^**^	0.2582^***^	0.0882
	(0.0355)	(0.0546)	(0.0538)	(0.0633)	(0.0500)	(0.0853)
Household income	0.0081^***^	0.0125^***^	0.0020	0.0077	0.0162^***^	0.0135^*^
	(0.0030)	(0.0044)	(0.0044)	(0.0054)	(0.0042)	(0.0071)
Employment status	0.0768^***^	−0.0461	0.0279	0.0059	0.0686^**^	−0.0002
	(0.0273)	(0.0341)	(0.0391)	(0.0462)	(0.0346)	(0.0681)
Residence	0.0318^***^	0.0255^**^				
	(0.0094)	(0.0129)				
Region			−0.0014	−0.0257	0.0771^***^	−0.0772
			(0.0308)	(0.0399)	(0.0277)	(0.0527)
_cons	1.3295^***^	1.3028^***^	1.6632^***^	1.1570^***^	1.2815^***^	1.1222^***^
	(0.1566)	(0.1407)	(0.1576)	(0.1687)	(0.1293)	(0.2410)
*N*	8,535	4,105	3,616	2,989	4,718	1,317
*R* ^2^	0.103	0.086	0.083	0.105	0.113	0.090
Adj. *R*^2^	0.101	0.083	0.079	0.101	0.110	0.079

Significance levels ^*^p <0.1; ^**^p <0.05; ^***^p <0.01.

The heterogeneity test results by region grouping demonstrate the impact of basic medical insurance integration on the subjective wellbeing of individuals from rural and West China is salient.

### Heterogeneity test of household characteristics

To test heterogeneity in the effects of integration on residents with different household characteristics, we stratify the analytic sample into three quartiles based on household income; individuals with the lowest 25% household income are low-income group, the middle-income quartile are individuals with 25–75% household income, and above the 75% quartile are defined as the high-income group. In addition, since the older adult are the most prevalent group of the disease, we also categorize individuals according to the presence or absence of a population aged 60 years and older in the household.

As shown in Table 6, the basic medical insurance integration significantly improves the subjective wellbeing of individuals from the middle-income group with a coefficient of 0.1846 (*p* < 0.05) indicating that the subjective wellbeing of the middle-income group is increased by the integration by 18.46%, while the positive effect on the low-income group and the high-income group is not significant.

**Table 6:** Heterogeneity test results for household characteristics.

	**(11)**	**(12)**	**(13)**	**(14)**	**(15)**
	**Low-income**	**Middle-income**	**High-Income**	**Household without the older adult aged 60 years and older**	**Household with the older adult aged 60 years and older**
Treat	−0.0754	−0.0163	0.0422	0.0228	−0.0579
	(0.0774)	(0.0554)	(0.0706)	(0.0484)	(0.0623)
Post	0.3077^***^	0.2765^***^	0.1696^***^	0.1934^***^	0.3735^***^
	(0.0431)	(0.0252)	(0.0312)	(0.0237)	(0.0255)
Did	0.0734	0.1846^**^	0.0593	0.0517	0.1955^**^
	(0.1144)	(0.0777)	(0.1013)	(0.0736)	(0.0824)
Age	0.0094^***^	0.0089^***^	0.0071^***^	0.0067^***^	0.0120^***^
	(0.0016)	(0.0013)	(0.0018)	(0.0013)	(0.0011)
Gender	−0.0612	0.0435	0.0160	0.0216	−0.0153
	(0.0499)	(0.0351)	(0.0429)	(0.0328)	(0.0357)
Education level	−0.0212	0.0041	−0.0185	0.0097	−0.0122
	(0.0332)	(0.0225)	(0.0311)	(0.0210)	(0.0246)
Political organization membership	0.0911^*^	0.0505	0.1565^***^	0.1185^***^	0.0709^**^
	(0.0517)	(0.0323)	(0.0391)	(0.0316)	(0.0322)
Hukou	0.0768	0.0939	0.0912	0.0816^*^	0.0785
	(0.0967)	(0.0571)	(0.0559)	(0.0457)	(0.0601)
Marriage status	0.2327^***^	0.0600	0.1974^***^	0.2418^***^	0.0788^**^
	(0.0581)	(0.0402)	(0.0564)	(0.0461)	(0.0365)
Self–assess health	0.1939^***^	0.1773^***^	0.1899^***^	0.2138^***^	0.1569^***^
	(0.0179)	(0.0119)	(0.0165)	(0.0116)	(0.0124)
Household size	0.0021	0.0023	0.0013	0.0018	−0.0023
	(0.0021)	(0.0015)	(0.0065)	(0.0014)	(0.0015)
Proportion of population aged 60 years and older	0.1220^*^	0.2063^***^	0.1826^***^		
	(0.0662)	(0.0414)	(0.0581)		
Employment status	0.0602	0.0493	−0.0924^**^	0.0309	0.0430
	(0.0402)	(0.0302)	(0.0449)	(0.0328)	(0.0285)
Residence	−0.0218	0.0410^*^	0.0306	0.0248	0.0120
	(0.0380)	(0.0244)	(0.0316)	(0.0224)	(0.0269)
Region	0.0163	0.0375^***^	0.0376^***^	0.0246^**^	0.0375^***^
	(0.0161)	(0.0110)	(0.0137)	(0.0101)	(0.0115)
Household income				0.0085^***^	0.0126^***^
				(0.0030)	(0.0044)
_cons	1.2590^***^	1.3051^***^	1.5707^***^	1.2405^***^	1.2034^***^
	(0.1738)	(0.1178)	(0.1561)	(0.1126)	(0.1314)
*N*	3,160	6,320	3,160	6,867	5,773
*R* ^2^	0.092	0.100	0.088	0.086	0.104
Adj. *R*^2^	0.087	0.098	0.084	0.084	0.102

Significance levels ^*^p <0.1; ^**^p <0.05; ^***^p <0.01.

In addition, the integration of basic medical insurance significantly increased the subjective wellbeing of individuals whose households have older adult aged 60 years and older by 12.78%; conversely, for households without the older adult, the coefficient is positive, too, but it is not significant.

## Discussion

We employed the PSM-DID method using CHFS2015 and CHFS2019 datasets to examine the impact of basic medical insurance integration on residents' subjective wellbeing in China. Our findings indicate a significant improvement in residents' subjective wellbeing following the implementation of basic medical insurance integration in 2016. The robustness of these results was confirmed through 1,000 Bootstrap placebo tests, which involved replacing matching methods (as shown in the Supplementary files). Our primary research finding highlights the positive effect of basic medical insurance integration on the subjective wellbeing of insured individuals. Moreover, the heterogeneity test revealed that the integration specifically benefited disadvantaged groups in underdeveloped areas of China, and residents with older adult dependents. Despite the expectation that the low-income group would benefit significantly from the implementation of the integration, it is the middle-income group that has experienced a notable enhancement in their subjective wellbeing.

### Analysis of the main finding

In the present study, our main finding highlights the positive impact of basic medical insurance integration on the subjective wellbeing of Chinese residents. This finding is consistent with the policy objectives of the country to improve the subjective wellbeing of its residents. The result is in line with the research conducted by Liu and Ling, who observed that basic medical insurance contributes to increased life satisfaction and a sense of social justice among residents (44). Their findings suggest that the basic medical insurance positively influences residents' subjective wellbeing and supports the rationale for implementing basic medical insurance integration to a certain extent.

Furthermore, this finding adds to the previous findings by further confirming the positive relationship between basic medical insurance integration and subjective wellbeing of the general population. Liu et al.'s study demonstrated that the integration not only significantly enhances positive affect among older adult individuals residing in rural areas but also effectively reduces negative affect, indicating an overall improvement in the subjective wellbeing of the older adult in rural areas (20). Our study similarly discovered the positive impact of basic medical insurance integration on subjective wellbeing; however, our study encompassed the entire population.

### Analysis of regional heterogeneity

The results of the heterogeneity test show that the impact of the integration on people's subjective wellbeing differs on the dimension of the region, with the effect of basic medical insurance integration being significantly positive on improving the subjective wellbeing of individuals from West China, while the positive effect is not significant on individuals from other regions. The overall socioeconomic development of West China is relatively inadequate, where residents are more vulnerable to illness or accidents as well as more likely to be influenced by policy benefits. The findings reported here are partly consistent with those reported by Cheng and Zhang, who investigated the impact of social pension insurance on the subjective wellbeing of residents using data from the China Family Panel Studies (CFPS). Cheng and Zhang found that subjective wellbeing was significantly enhanced by the social pension insurance for residents from both East China and West China, with the former having greater life satisfaction than the latter. They held that the implementation of social pension insurance varied across the country, and East China had better economic development and a higher degree of acceptance of social insurance policies, meaning that residents in this region have less financial pressure and higher current life satisfaction with social insurance implementation (45). Considering Cheng and Zhang focused on social pension insurance, while we concentrate on basic medical insurance, we assume that residents from relatively less developed West China need to overcome financial pressure to purchase social pension insurance, which is a long-term investment requiring residents to pay contributions since their young-age and the pension will provide a stable source of income and livelihood security when they get old. By contrast, basic medical insurance is a short-term investment that residents get reimbursement in a fiscal year, so it enhances the subjective wellbeing of residents from West China rather than putting the financial burden on residents.

Liu et al., by comparison, who analyzed data from the China Health and Retirement Longitudinal Study (CHARLS), found no evidence of improvement in the subjective wellbeing of residents from West China as a result of basic medical insurance integration (20). They believed that the medical resources in West China were relatively insufficient resulting in limited utilization of the URRBMI, hence little improvement in the subjective wellbeing of residents from West China. The differences between our findings and their findings may be explained in terms of the difference in databases used and the subject included. The study of Liu et al. mainly focused on the rural older adult, while our study employed a nationally representative covering the entire population. As Liu et al. mentioned, the inadequate medical resource in West China might be an obstacle for rural older adult to benefit from the URRBMI; since we found that the subjective wellbeing of the entire population from West China was enhanced, we assume that young residents benefit more from the URRBMI than the older adult, which may be due to not providing sufficient coverage for common illnesses of the older adult, such as chronic diseases.

Similarly, the heterogeneity test of regional characteristics also shows that the basic medical insurance integration contributes to enhancing rural residents' subjective wellbeing, while improving urban residents' subjective wellbeing insignificantly. Similar comments apply to the study by Chang et al., who analyzed data from the China Labor-force Dynamic Survey (CLDS) and found that the implementation of the URRBMI in rural areas had a significant positive effect on increasing healthcare expenditures, implying that rural residents increased their utilization of medical services after the integration, while the integration did not improve medical expenditures of urban residents. Chang et al. explained that the demand for medical services of rural residents was suppressed due to limited benefit packages before the integration, while this is not the case for urban residents (16). With a higher reimbursement rate and annual cap, as well as more comprehensive coverage of insured drugs and medical services, the URRBMI facilitates rural residents covered by the NRCMS before utilizing healthcare services efficiently, while it does not affect urban residents insured by the URBMI of which the benefit packages are comparable with the URRBMI. Hence, the integration improves the subjective wellbeing of rural residents correspondingly, whereas it does not impact urban residents.

### Analysis of household heterogeneity

On the dimension of household, the results of the heterogeneity test indicate that the incentive effect of the integration is significant for individuals who have older adult family members aged 60 years and over, but not for those without the older adult to support. The potential reason for this is that the older adult are prone to illness, hence, exposure to health risks, and compared with the NRCMS and URBMI, URRBMI has a higher reimbursement rate, which further relieves the medical burden of families with senior citizens and enhances their sense of wellbeing (46).

At the same time, the positive effect is significant for the individuals from middle-income families, but not for the ones from low-income and high-income families. And we give the following explanations from the perspectives of the low-income group and high-income group.

According to Maslow's hierarchy of needs, esteem, friendship and love, security, and physical needs are deficiency needs for the individual from higher status to lower status, and the most basic level of needs must be met before the individual will strongly desire (or focus motivation upon) the secondary or higher-level needs. In other words, low-income groups are more likely to concern about shelter, health, financial security, and other physical and security needs, hence one of the expected policy goals of basic medical insurance integration reduces the probability of falling into poverty caused by illness for low-income residents, so the effect of basic medical insurance on spiritual happiness is more limited for the low-income group (47). Moreover, residents paid 120 CNY per person per year for NRCMS in 2015 (48), while it was 320 CNY per person per year for URRBMI in 2021 (49), which is an undoubtedly increasing burden for low-income people which limits the improvement of their subjective wellbeing.

While in terms of the elasticity of demand for healthcare services, the high-income group has less elasticity of demand than low- and middle-income groups (16), i.e., the high-income group is less sensitive to the financial burden brought by basic medical insurance integration and is less affected by insurance than other groups.

While Liu et al. yielded different results in the study of how integration impacts the subjective wellbeing of rural older adult, they found that the integration improved the subjective wellbeing of low-, middle-, and high-income groups, in turn, showing a pro-rich effect. They argued that China had an imbalanced allocation of medical resources, with high-income people often enjoying more and low-income people having less access to medical resources (20). Considering that the subjective wellbeing of the high-income group was not increased by the integration as we included the entire population in our study, it can be inferred that the integration may have a negative effect on improving the subjective wellbeing of the high-income population except the rural older adult so that the positive effect of the integration on high-income group found by Liu et al. with a focus on rural older adult is offset.

In addition, the findings of Chang et al. may explain part of the reasons that the integration cannot improve the subjective wellbeing of the low-income group. Chang et al. found that the implementation of the URRBMI stimulated the demand of low-income groups for healthcare services and increased their medical expenses of them (16). Therefore, to our mind, the integration led to a greater financial burden of healthcare services and less improvement of subjective wellbeing among low-income residents.

### Practical implications

Although the above evidence validate that the basic medical insurance integration enhances the subjective wellbeing of the general population, it does not account for the vulnerable low-income residents who should benefit from the URRBMI. Hence, we propose the following policy recommendations from the perspectives of funding and utilization of the URRBMI.

On the one hand, a dynamic adjustment funding system that associates residents' medical insurance funding standard with their disposable income may be more appropriate for China's basic medical schemes, so that the benefits of high-income insured individuals can be increased while the burden of low-income ones can be lifted. The insured residents meet their expectations efficiently under the current defined contribution funding system, but it is unable to personalize the contributions of different populations in the same region. Given that China has not implemented the self-declaration of individual income completely, it is challenging to connect the medical insurance funding standard with personal income (50). However, with the improvement of the income declaration system and tax information system, the changes in residents' disposable income will be grasped in time (51), and the correlation between contribution and self-declared income will be realized. Furthermore, under the dynamic adjustment funding system, it will be easier to track changes in the income of the population who are vulnerable to health and financial risk, so that medical assistance can be provided as a supplement to basic medical insurance (52).

On the other hand, the benefit packages of the URRBMI should be enlarged, especially more outpatient services should be covered through risk pooling. The National Healthcare Security Administration of China issued a document in 2019 proposing that the URRBMI should cover outpatient services coordinately (53) since only the inpatient-insured residents could benefit from the URRBMI in the first place, which undermined the willingness of the majority to apply for the insurance, as well as resulted in adverse selection and moral hazard (54). In contrast, if the URRBMI reimburses outpatient services, the timeliness of care and utilization of outpatient services will be improved, so that the burden of residents' healthcare can be further relieved. Nevertheless, the current URRBMI reimbursement for outpatient is insufficient, especially since the annual caps in central and western regions are so low that do not match the average number of visits and average per-visit costs of residents (55). In addition, residents are encouraged to visit primary healthcare facilities through the URRBMI reimbursement policy for outpatient care to address the unbalanced allocation of healthcare resources and the underutilization of primary care facilities (56), but current service capacity and quality are suboptimal, that many township health centers in remote areas are not even capable of providing some common services (57). Therefore, improving the quality and capacity of the primary healthcare system of China merits consideration.

### Limitations and avenues for future research

Our findings should be understood in light of the following caveats.

First, the integration of basic medical insurance was implemented separately by provinces due to China's social health insurance's nature of fragmentation. Moreover, the benefit packages of basic medical insurance schemes varied across cities and even counties. Given the inclusion of heterogeneity tests for different regions, the overall findings are not affected as long as the implementation of the integration and the benefit packages of the basic medical insurance schemes are similar within a region where socioeconomic characteristics are comparable, although we cannot categorize population into province, city, or county level segments by virtue of data limitation. For future study, a refined grouping method, i.e., categorizing individuals by smaller administrative divisions, is recommended.

Second, our findings indicate only short-term effects of basic medical insurance integration on residents' subjective wellbeing, since the latest available data on CHFS is the one released in 2021 for 2019, hence, evaluation with more time observed is suggested for further study.

Finally, the sample size is not large enough. Only 563 individuals out of 12,654 respondents switched their insurance types in the 3rd year of the implementation of the basic medical insurance integration, since the previous medical insurance schemes and the URRBMI were ambiguous for the residents in the initial stage of the integration. Some respondents might choose the NRCMS and URBMI in the self-report survey, even if they were covered by the URRBMI in 2019. So, we suggest that researchers give respondents a detailed introduction to basic medical insurance.

## Conclusion

Our findings suggest that the basic medical insurance integration facilitates China's part of the policy goal that to pursue equality and universal benefits for residents (58). The implementation of the URRBMI improves the subjective wellbeing of the insured population, particularly benefits disadvantaged groups from less developed West China and rural areas, as well as residents with older adult dependents, while it improves the subjective wellbeing of middle-income groups rather than low-income groups who are supposed to benefit more from the URRBMI. Such experiences are worth being extended to countries and regions where basic medical insurance is still in fragmentation.

## Data availability statement

The data we analyzed in this study is publicly available datasets, which can be found in the CHFS repository: https://chfs.swufe.edu.cn/sjzx.htm.

## Author contributions

XN: conceptualization, writing—original draft, literature search, and interpretation of the findings. LD: conceptualization, writing—original draft, methodology, and software. JW: writing—reviewing and editing. SC: supervision. All authors contributed to the final version of the manuscript and agreed with the order of presentation of the authors.

## Footnotes

^1^National Bureau of Statistics of China/Annual/Public Management, Social Security and Others/Urban Basic Medical Care Insurance/Persons Covered at Year-end, https://data.stats.gov.cn/english/easyquery.htm?cn=C01.
