# Supplementary material for: Effects of basic medical insurance integration on subjective wellbeing of residents in China: empirical evidence from a quasi-experiment
Source: Front Public Health. 2023 Aug 16;11:1211350. doi: 10.3389/fpubh.2023.1211350 (PMC10467426; doi:10.3389/fpubh.2023.1211350)
Supplement: Supplementary file 1 [file Data_Sheet_1.docx]

# Supplementary files—robustness test

## Replacement of matching method

The analysis above validates the positive incentives for basic medical insurance integration on residents' subjective well-being. Since we use the 1:1 nearest neighbor matching for propensity score matching, if we replace the previous matching methods with other matching strategy and obtain the similar results, it can corroborate the robustness of our findings. Therefore, we further conduct the DID regression analysis based on kernel matching and caliper matching with higher matching accuracy, where the caliper distance is 0.01. As shown in Table 7, Models (16) and (17) report the DID estimates after kernel matching and caliper matching, respectively. The results show that the net effects of both models are positive at the 5% significance level and the regression coefficients are similar in a large extent, indicating that basic medical insurance integration has a significant improvement effect on residents’ subject well-being, which is consistent with the previous findings and proves the robustness of our results.

Table 7 Results of DID based on kernel matching and caliper matching

|  | (16) | (17) |
| --- | --- | --- |
|  | Kernel matching | Caliper matching |
| Treat | -0.0105 | -0.0113 |
|  | (0.0378) | (0.0378) |
| Post | 0.2667^***^ | 0.2645^***^ |
|  | (0.0175) | (0.0174) |
| Did | 0.1247^**^ | 0.1255^**^ |
|  | (0.0534) | (0.0534) |
| Age | 0.0090^***^ | 0.0089^***^ |
|  | (0.0009) | (0.0009) |
| Gender | 0.0157 | 0.0183 |
|  | (0.0232) | (0.0232) |
| Education level | -0.0013 | 0.0027 |
|  | (0.0160) | (0.0160) |
| Political organization membership | 0.0907^***^ | 0.0964^***^ |
|  | (0.0246) | (0.0244) |
| Hukou | -0.0775^**^ | -0.0800^**^ |
|  | (0.0377) | (0.0377) |
| Marriage status | -0.0822^***^ | -0.0803^***^ |
|  | (0.0254) | (0.0254) |
| Self-assess health | 0.1872^***^ | 0.1886^***^ |
|  | (0.0079) | (0.0079) |
| Household size | 0.0014 | 0.0015 |
|  | (0.0010) | (0.0010) |
| Proportion of population aged 60 years and older | 0.1650^***^ | 0.1652^***^ |
|  | (0.0294) | (0.0294) |
| Household income | 0.0106^***^ | 0.0095^***^ |
|  | (0.0025) | (0.0024) |
| Employment status | 0.0419^**^ | 0.0393^*^ |
|  | (0.0203) | (0.0203) |
| Residence | 0.0133 | 0.0154 |
|  | (0.0173) | (0.0173) |
| Region | 0.0310^***^ | 0.0295^***^ |
|  | (0.0078) | (0.0077) |
| _cons | 1.6800^***^ | 1.6872^***^ |
|  | (0.0973) | (0.0969) |
| *N* | 12550 | 12643 |
| *R*^2^ | 0.095 | 0.094 |
| adj. *R*^2^ | 0.093 | 0.093 |
| Note: Significance levels *p < 0.1; **p < 0.05; ***p < 0.01. | | |

## Placebo test

Referring to existing literature, we conduct a placebo test by randomly selecting treatment groups from the sample to investigate whether there are unobserved random factors influence the policy effects of basic medical insurance integration on residents’ subjective well-being^[[1]](#footnote-1)^. We randomly select individuals from the sample as the pseudo group based on the 563 individuals that switch their basic medical insurance type in the integration, and the interaction term for pseudo is generated based on the time dummy variable. We repeat the regressions for 1000 times with residents' subjective well-being as the explained variable, then make the kernel density estimation of the estimators and t-values of the policy effects as well as present the results in Figure 2, where reports the distribution of the estimated coefficients and the corresponding t-values of the interaction terms. Figure 2 shows that the estimated coefficients of the interaction terms of the pseudo group during the 1000 random treatments are concentrated around 0, so are the t-values, and this manifests that we do not obtain previous results by chance and verifies the robustness of our empirical study.


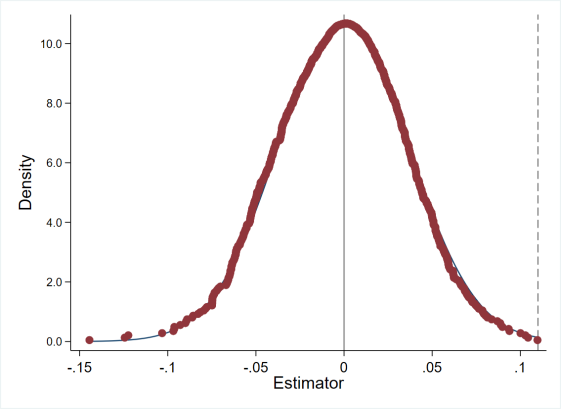

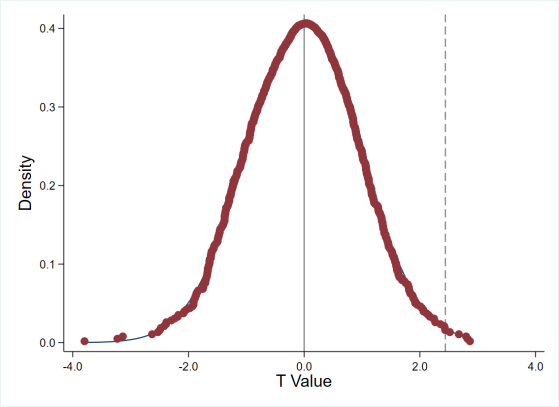


(A) (B)

Figure 2 kernel density curves of estimators and t-values

1. Cantoni D, Chen Y, Yang DY, Yuchtman N, Zhang YJ. Curriculum and ideology. *Journal of political economy* (2017) 125(2):55. doi:10.1086/690951 [↑](#footnote-ref-1)
